# Association Between Vitamin D Deficiency and Systemic Outcomes in Patients with Glaucoma: A Real-World Cohort Study

**DOI:** 10.3390/nu18020261

**Published:** 2026-01-14

**Authors:** Shan-Shy Wen, Chien-Lin Lu, Ming-Ling Tsai, Ai-Ling Hour, Kuo-Cheng Lu

**Affiliations:** 1 Graduate Institute of Applied Sciences and Engineering, Fu Jen Catholic University, New Taipei City 242062, Taiwan; 136811@mail.fju.edu.tw; 2 Division of Nephrology, Department of Internal Medicine, Fu Jen Catholic University Hospital, Fu Jen Catholic University, New Taipei City 243089, Taiwan; 096195@mail.fju.edu.tw; 3 School of Medicine, College of Medicine, Fu Jen Catholic University, New Taipei City 242062, Taiwan; 4 Department of Ophthalmology, Taipei Buddhist Tzu Chi Hospital, Buddhist Tzu Chi Medical Foundation, New Taipei City 231016, Taiwan; doc30845@yahoo.com.tw; 5 Department of Life Science, Fu Jen Catholic University, New Taipei City 242062, Taiwan; 6 Division of Nephrology, Department of Medicine, Taipei Tzu Chi Hospital, Buddhist Tzu Chi Medical Foundation, New Taipei City 231016, Taiwan

**Keywords:** vitamin D deficiency, glaucoma, cardiorenal outcomes, acute kidney injury, all-cause mortality

## Abstract

**Background:** Glaucoma is an age-related optic neuropathy frequently accompanied by systemic comorbidities. Vitamin D deficiency (VDD) has been associated with cardiovascular and renal diseases in the general population, yet its relationship with long-term systemic outcomes in glaucoma remains unclear. This study evaluated the association between baseline vitamin D status and subsequent mortality and cardiorenal events in patients with primary glaucoma. **Methods:** We conducted a retrospective cohort study using deidentified electronic health records from the TriNetX U.S. Collaborative Network, a federated network of participating healthcare organizations. Adults (≥18 years) with incident primary glaucoma (2005–2020) and a serum 25-hydroxyvitamin D (25(OH)D) test within 12 months prior to diagnosis were categorized as VDD (<30 ng/mL) or vitamin D adequacy (VDA; ≥30 ng/mL). After 1:1 propensity score matching across 47 demographic, clinical, medication, and laboratory variables, 11,855 patients per group were followed for up to 5 years. Outcomes included all-cause mortality, major adverse cardiovascular events (MACE), acute kidney injury (AKI), and renal function decline (eGFR < 60 mL/min/1.73 m^2^). Analyses incorporated Kaplan–Meier curves, Cox models, landmark tests, sensitivity analyses, and competing risk methods. **Results**: Among the 35,100 eligible patients, the matched cohorts demonstrated higher 5-year risks associated with VDD for all-cause mortality (HR 1.104; 95% CI 1.001–1.217), MACE (HR 1.151; 95% CI 1.078–1.229), and AKI (HR 1.154; 95% CI 1.056–1.261), whereas the risks of renal function decline did not differ (HR 0.972; 95% CI 0.907–1.042). Risk divergence emerged within the first year of follow-up and persisted through the 5-year observation period. **Conclusions**: In patients with primary glaucoma, vitamin D deficiency was associated with higher long-term risks of mortality and cardiorenal complications, but not renal function decline. Taken together, the results are consistent with vitamin D status serving as a marker of broader systemic vulnerability in glaucoma and highlight the need for prospective studies to further clarify its prognostic significance.

## 1. Introduction

Glaucoma is a leading cause of irreversible blindness worldwide, affecting an estimated 76.0 million people in 2020 and projected to reach 111.8 million by 2040. It predominantly affects older adults, many of whom have chronic comorbidities such as hypertension, diabetes, and chronic kidney disease [1,2,3]. Although glaucoma is traditionally characterized by progressive optic neuropathy and intraocular pressure dysregulation, accumulating evidence indicates that systemic vascular, metabolic, and inflammatory pathways also contribute to disease development and progression [4,5,6]. These shared mechanisms suggest that glaucoma may be a clinical manifestation of broader systemic vulnerability, which is associated with increased morbidity and mortality.

Clinically, understanding systemic health risks among patients with glaucoma remains challenging. Standard ophthalmic parameters—such as intraocular pressure, visual field loss, and optic nerve morphology—provide limited information regarding overall physiological vulnerability. Glaucoma patients frequently exhibit heterogeneous cardiovascular and renal profiles, reflecting diverse systemic conditions that may influence both ocular and extraocular outcomes [7,8,9]. However, the extent to which these systemic disturbances affect long-term health trajectories in glaucoma is not fully understood. Defining these relationships may improve recognition of glaucoma as a multisystem disorder and guide preventive and inform risk awareness and interdisciplinary care considerations in an aging population.

Vitamin D plays a key role in systemic health. Beyond its established function in calcium homeostasis, 1,25-dihydroxyvitamin D exerts broad regulatory effects on vascular endothelial integrity, immune modulation, oxidative stress, and neuroprotection through activation of the vitamin D receptor (VDR) across multiple tissues, including the retina and optic nerve head [10,11,12,13]. Vitamin D deficiency (VDD) has been consistently linked to cardiovascular disease, renal dysfunction, and all-cause mortality in the general population, suggesting that inadequate vitamin D status may be a marker of broader systemic dysregulation relevant to aging and chronic disease [14,15,16,17]. Notably, accumulating evidence from large observational studies and randomized controlled trials indicates that vitamin D deficiency is more likely a risk marker of poor systemic health or frailty rather than a direct causal determinant of adverse clinical outcomes [18,19,20].

Evidence linking vitamin D status to glaucoma has been heterogeneous and largely observational. Several cross-sectional and case–control studies report that patients with glaucoma tend to have lower serum 25-hydroxyvitamin D levels than controls, suggesting a possible association between VDD and increased glaucoma risk in specific demographic groups [21,22]. However, findings remain inconsistent: other studies show no significant differences in vitamin D levels between glaucoma and non-glaucoma populations, nor any relationship with disease severity or progression [23,24,25]. Longitudinal and Mendelian randomization analyses likewise provide no causal evidence that genetically determined vitamin D levels influence the risk or progression of primary open-angle glaucoma [26,27]. Although VDD has been implicated in neurodegenerative, cardiovascular, and metabolic disorders [22,28,29], no prior studies have directly examined whether vitamin D status affects long-term systemic outcomes in patients with glaucoma. Taken together, the existing literature suggests at most a modest association—and no established causal relationship—highlighting the need for large-scale longitudinal research.

To address this knowledge gap, we conducted a large real-world cohort study using the TriNetX platform, with analyses performed within the U.S. Collaborative Network, to investigate the longitudinal association between baseline vitamin D status and major systemic outcomes—including all-cause mortality, major adverse cardiovascular events (MACE), and acute kidney injury (AKI)—in patients with primary glaucoma. We hypothesized that VDD would be associated with higher risks of mortality and cardiorenal events, consistent with its role as a non-causal marker of systemic vulnerability in patients with glaucoma. Clarifying this association may improve understanding of glaucoma as a multisystem condition and support more integrated, preventive approaches to care in this complex population.

## 2. Methods

### 2.1. Study Design and Data Source

This retrospective cohort study was conducted using deidentified patient-level data obtained from the TriNetX platform, a federated global health research network that integrates real-world electronic health records (EHRs) from hospitals and healthcare systems across multiple geographic regions, including North America, Europe, Asia-Pacific, and Latin America. The analysis was executed on 11 October 2025, within the secure TriNetX cloud-based analytic environment using standardized federated queries. The TriNetX platform enables distributed analyses across contributing healthcare organizations while maintaining data deidentification and local data governance. The database contains comprehensive longitudinal clinical information, including demographics, diagnostic codes, procedures, medication prescriptions, and laboratory results.

All data extraction and analyses were performed within the secure TriNetX cloud environment. Although the data provided were fully deidentified in compliance with the Health Insurance Portability and Accountability Act (HIPAA) and the General Data Protection Regulation (GDPR), the study protocol still underwent institutional ethical review. All study procedures adhered to the ethical principles outlined in the Declaration of Helsinki. The full study protocol was reviewed and approved by the Taipei Tzu Chi Hospital Institutional Review Board, under approval number 14-IRB134.

### 2.2. Study Population and Inclusion Criteria

The source population consisted of adult patients aged 18 years or older with a first recorded diagnosis of primary open-angle glaucoma or primary angle-closure glaucoma recorded between 1 January 2005 and 1 January 2020. Glaucoma diagnoses were identified using the International Classification of Diseases, Tenth Revision, Clinical Modification (ICD-10-CM) codes H40.xx–H42.xx. To enhance diagnostic specificity, patients with secondary glaucoma—due to ocular trauma, inflammation, other eye disorders, or medications (H40.3–H40.6)—were excluded. The index diagnosis represented the earliest date on which a qualifying primary glaucoma code was documented. To define baseline vitamin D exposure, inclusion additionally required at least one serum 25-hydroxyvitamin D [25(OH)D] measurement (LOINC code 1989-3) obtained within the 12 months preceding the index glaucoma diagnosis. When multiple measurements were available within this window, the value closest to the index date was selected to represent baseline vitamin D status. Patients were classified into exposure groups based on this most recent value: vitamin D deficiency (VDD) defined as 25(OH)D < 30 ng/mL and vitamin D adequacy (VDA) defined as 25(OH)D ≥ 30 ng/mL. After applying the eligibility criteria, 17,865 patients were included in the VDD group and 17,235 in the VDA group. To enhance exposure stability and reduce misclassification, individuals in the VDA cohort had no prior 25(OH)D measurements < 30 ng/mL during the enrollment period, while those in the VDD cohort had no prior measurements ≥ 30 ng/mL, thereby excluding patients with recent vitamin D status transitions. In this study, the term “vitamin D adequacy” (VDA) was used solely as an analytical label to denote a clearly replete reference group for comparison, rather than to imply an optimal clinical target or therapeutic recommendation.

### 2.3. Index Date and Follow-Up

The index date was defined as the date of the first qualifying diagnosis of primary glaucoma that satisfied all inclusion criteria. Follow-up commenced on the day after the index date to ensure proper temporal separation between exposure assessment and outcomes ascertainment. The observation period continued until the earliest occurrence of 1825 days (5 years), death, loss to follow-up, or the end of available electronic health records. Patients who had evidence of a study outcome prior to the follow-up window were automatically excluded from the analysis of that specific outcome to prevent misclassification and ensure accurate capture of incident events.

### 2.4. Outcome Measures

The primary outcome was all-cause mortality, determined by a documented deceased status in structured demographic fields; ICD-10-CM code R99 was used only as a supportive indicator when structured mortality fields were unavailable, acknowledging potential misclassification. Secondary outcomes included major adverse cardiovascular events (MACE), acute kidney injury (AKI), and decline in renal function defined by an estimated glomerular filtration rate (eGFR) < 60 mL/min/1.73 m^2^. MACE was defined as a composite outcome of acute myocardial infarction, cardiac arrest, heart failure, nontraumatic intracerebral hemorrhage, cerebral infarction, ischemic heart disease, or death. AKI was identified using the ICD-10-CM code N17. Renal function decline was evaluated using the most recent eGFR obtained during follow-up, calculated using the MDRD equation as implemented on the TriNetX platform. All outcome definitions followed standardized TriNetX terminology and were uniformly applied across cohorts to ensure consistent ascertainment.

### 2.5. Propensity Score Matching and Handling of Confounders

To reduce baseline imbalances and potential confounding, 1:1 propensity score matching (PSM) was performed using a greedy nearest-neighbor algorithm without replacement. Matching incorporated 47 clinically relevant variables, including demographic characteristics, cardiometabolic comorbidities, cardiovascular and antidiabetic medications, renal and metabolic laboratory indices, electrolyte parameters, hematologic profiles, and inflammatory biomarkers. Standardized mean differences (SMDs) were used to assess covariate balance, with SMD < 0.1 considered indicative of acceptable comparability. After matching, each exposure cohort comprised 11,855 patients, with marked improvement in baseline covariate balance, allowing more reliable estimation of the association between vitamin D status and outcomes. Matching was conducted using available-case analysis as implemented by the TriNetX platform, with no imputation for missing values; post-matching standardized mean differences (SMD < 0.1) verified balance across covariates (Table 1).

### 2.6. Statistical Analyses

Time-to-event analyses were performed using Kaplan–Meier curves with log-rank testing. Cox proportional hazards models were applied to estimate hazard ratios with 95% confidence intervals for all clinical outcomes. Patients who experienced an outcome before the start of follow-up were excluded to ensure incident event capture. A two-sided *p*-value < 0.05 was considered statistically significant. All analyses were conducted within the secure TriNetX cloud environment using its standardized analytic pipelines.

### 2.7. Sensitivity Analyses

To evaluate the robustness of the primary outcome findings, two sensitivity analyses were performed (Supplementary Tables S1 and S2). First, to assess whether the observed associations between VDD and VDA were influenced by baseline cohort imbalance, outcomes were compared both before and after propensity score matching (Supplementary Table S1). Second, to examine whether the association varied by the severity of vitamin D deficiency, an alternative exposure threshold of 25(OH)D < 20 ng/mL was applied in addition to the primary < 30 ng/mL definition (Supplementary Table S2). Hazard ratios, 95% confidence intervals, and log-rank *p*-values were reported to maintain consistency with the main analytic framework.

Additional analyses included negative control outcome analyses and E-value sensitivity analyses to assess residual confounding (Supplementary Tables S3 and S4), exclusion of patients with major acute illnesses around the time of vitamin D measurement to reduce reverse causality (Supplementary Table S5), restricted propensity score matching using covariates with high data completeness to evaluate matching stability (Supplementary Table S6), and healthcare utilization analyses to contextualize potential outcome misclassification and surveillance bias (Supplementary Table S7).

## 3. Results

### 3.1. Patient Identification, Baseline Characteristics, and Propensity Score Matching

**Figure 1:**
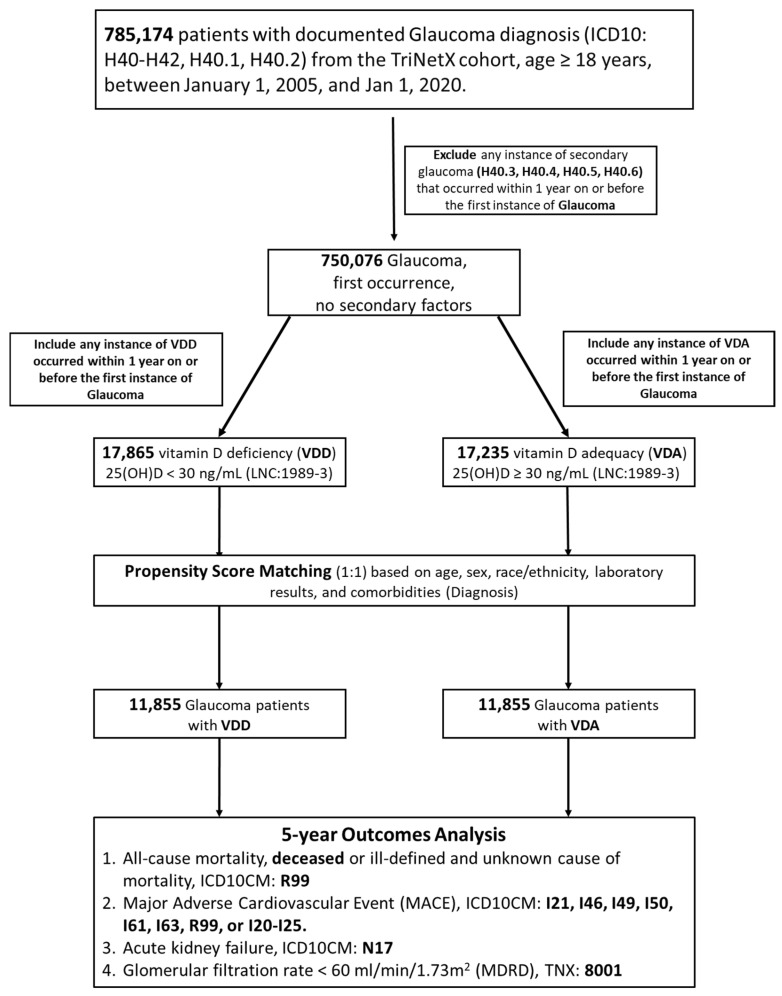
Flowchart of cohort selection. Adult patients with a documented diagnosis of primary glaucoma were identified from the TriNetX U.S. Collaborative Network between 2005 and 2020. After sequential exclusion of secondary glaucoma etiologies and classification based on serum 25(OH)D measured within 1 year prior to diagnosis, patients were categorized into vitamin D deficiency (VDD) and vitamin D adequacy (VDA) groups. Following 1:1 propensity score matching, 11,855 patients in each group were retained for comparative 5-year outcome analyses.

A total of 785,174 adult patients aged ≥ 18 years with a documented glaucoma diagnosis between 1 January 2005 and 1 January 2020 were identified from the TriNetX U.S. Collaborative Network. After excluding patients with secondary glaucoma etiologies to ensure a cohort representing primary glaucoma, 750,076 eligible individuals remained. Within this population, serum 25-hydroxyvitamin D measurements obtained within one year prior to the index glaucoma diagnosis were used to classify patients into two exposure groups: 17,865 individuals with VDD (25[OH]D < 30 ng/mL) and 17,235 individuals with VDA (≥30 ng/mL). As shown in Figure 1, the two groups were subsequently balanced using 1:1 propensity score matching based on demographics (age, sex, race/ethnicity), comorbid diagnoses, and relevant laboratory parameters, yielding two well-matched cohorts comprising 11,855 patients each.

Before matching, substantial differences in demographic, cardiometabolic, and biochemical characteristics were observed between VDD and VDA groups; however, after matching, covariates were well balanced with negligible standardized differences across all variables—except for serum calcidiol, which remained distinct by design—as shown in Table 1. The matched cohorts were then followed for up to five years after the index date to evaluate the comparative incidence of all-cause mortality, MACE, AKI, and renal function decline defined as an eGFR < 60 mL/min/1.73 m^2^ (calculated using the MDRD formula).

### 3.2. Primary Outcomes: Kaplan–Meier Survival Analysis

**Figure 2:**
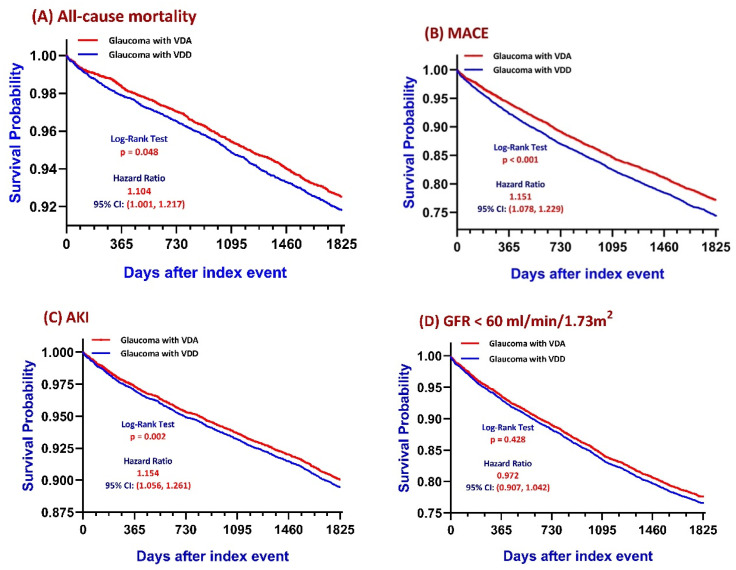
Kaplan–Meier Time-to-Event Curves for 5-Year Clinical Outcomes. Kaplan–Meier curves depict the cumulative incidence of all-cause mortality, major adverse cardiovascular events (MACE), acute kidney injury (AKI), and renal function decline (eGFR < 60 mL/min/1.73 m^2^) over a 5-year follow-up period among patients with vitamin D deficiency (VDD) and vitamin D adequacy (VDA). Differences between groups were evaluated using log-rank tests and Cox proportional hazards models.

Kaplan–Meier analyses revealed differential risks across major clinical outcomes between glaucoma patients with VDD and those with VDA over the 5-year follow-up period (Figure 2). VDD was associated with a higher risk of all-cause mortality, with a lower survival probability throughout the observation window (log-rank *p* = 0.048) and an elevated hazard of death (HR = 1.104; 95% CI, 1.001–1.217) (Figure 2A). A comparable elevation in risk was observed for MACE, where the VDD group experienced a significantly higher cumulative incidence than the VDA group (log-rank *p* < 0.001; HR = 1.151; 95% CI, 1.078–1.229) (Figure 2B). The incidence of acute kidney injury (AKI) was likewise higher in the VDD group (log-rank *p* = 0.002; HR = 1.154; 95% CI, 1.056–1.261) (Figure 2C). By contrast, no statistically significant difference was observed in renal function decline, defined as an eGFR <60 mL/min/1.73 m^2^ (log-rank *p* = 0.428; HR = 0.972; 95% CI, 0.907–1.042) (Figure 2D).

### 3.3. Landmark Analyses of Time-Dependent Outcome Differences

Landmark analyses at 1, 3, and 5 years following the index diagnosis were performed to evaluate time-dependent differences between the VDD and VDA groups (Table 2). At the 1-year landmark, VDD was associated with significantly higher risks of all-cause mortality (HR = 1.306; 95% CI, 1.066–1.600; *p* = 0.010), MACE (HR = 1.278; 95% CI, 1.127–1.449; *p* < 0.001), and AKI (HR = 1.288; 95% CI, 1.081–1.534; *p* = 0.004), while no difference was observed in renal function decline (*p* = 0.993). At the 3-year landmark, the elevated risk for MACE persisted (HR = 1.172; 95% CI, 1.080–1.272; *p* < 0.001), whereas the associations for all-cause mortality (HR = 1.132; 95% CI, 0.998–1.284; *p* = 0.053) and AKI (HR = 1.113; 95% CI, 0.995–1.246; *p* = 0.061) were borderline. By 5 years, VDD remained significantly associated with higher risks of all-cause mortality (HR = 1.104; 95% CI, 1.001–1.217; *p* = 0.048), MACE (HR = 1.151; 95% CI, 1.078–1.229; *p* < 0.001), and AKI (HR = 1.154; 95% CI, 1.056–1.261; *p* = 0.002), with no significant difference in the incidence of renal function decline (HR = 0.972; 95% CI, 0.907–1.042; *p* = 0.428).

### 3.4. Subgroup and Effect Modification Analyses

**Figure 3:**
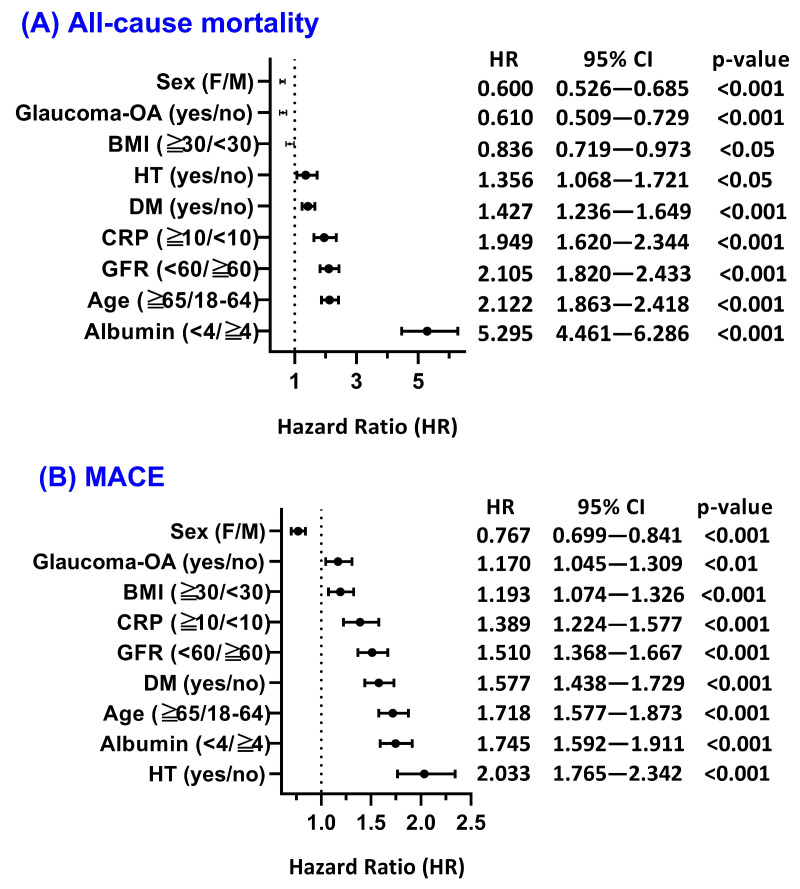

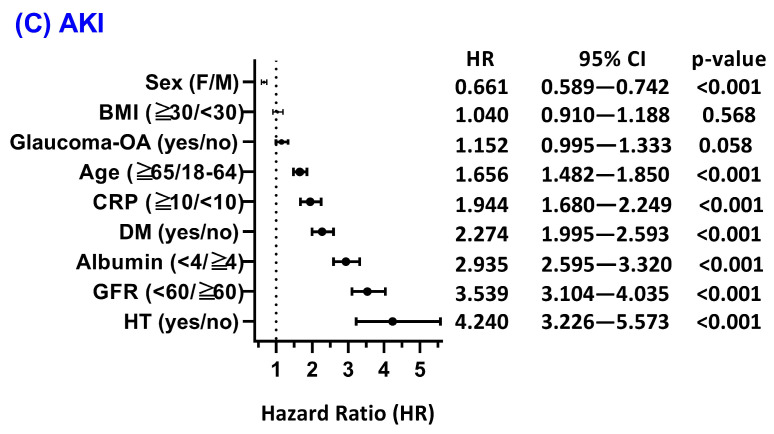
Subgroup hazard ratio analysis for 5-year adverse outcomes in glaucoma patients. Forest plots display hazard ratios (HRs) with 95% confidence intervals for key clinical subgroups associated with (**A**) all-cause mortality, (**B**) major adverse cardiovascular events (MACE), and (**C**) acute kidney injury (AKI). Subgroup effects were estimated using Cox proportional hazards models in the matched cohorts, demonstrating that systemic comorbidities—particularly impaired renal function, hypoalbuminemia, hypertension, diabetes, and advanced age—were the predominant predictors of long-term adverse outcomes. Glaucoma subtype was defined using diagnostic codes and should be interpreted as descriptive heterogeneity rather than a measure of disease severity, as detailed severity indicators were not available in the database.

Subgroup analyses were performed to explore whether the association between vitamin D status and clinical outcomes was modified by key demographic or clinical characteristics (Figure 3). Overall, systemic comorbidities exerted substantially greater influence on outcome risk than glaucoma subtype. Accordingly, subtype analyses should be interpreted as descriptive heterogeneity rather than as indicators of disease severity, as detailed glaucoma severity measures were not available in the database.

For all-cause mortality, hypoalbuminemia showed the most pronounced association (albumin < 4 vs. ≥4 g/dL: HR = 5.295; 95% CI, 4.461–6.286; *p* < 0.001). Older age (≥65 vs. 18–64 years: HR = 2.122; 95% CI, 1.863–2.418; *p* < 0.001) and reduced renal function (eGFR <60 vs. ≥60 mL/min/1.73 m^2^: HR = 2.105; 95% CI, 1.820–2.433; *p* < 0.001) were each associated with an approximately twofold higher risk. Elevated C-reactive protein (CRP) (HR = 1.949; 95% CI, 1.620–2.344; *p* < 0.001), diabetes (HR = 1.427; 95% CI, 1.236–1.649; *p* < 0.001), and hypertension (HR = 1.356; 95% CI, 1.068–1.721; *p* = 0.012) conferred moderate increases in mortality risk. Female sex demonstrated a protective association (HR = 0.600; 95% CI, 0.526–0.685; *p* < 0.001), while BMI ≥ 30 kg/m^2^ showed a modest inverse association (HR = 0.836; 95% CI, 0.719–0.973; *p* = 0.020).

For MACE, hypertension was the strongest predictor (HR = 2.033; 95% CI, 1.765–2.342; *p* < 0.001), followed by reduced renal function (HR = 1.510; 95% CI, 1.368–1.667; *p* < 0.001), diabetes (HR = 1.577; 95% CI, 1.438–1.729; *p* < 0.001), older age (HR = 1.718; 95% CI, 1.577–1.873; *p* < 0.001), hypoalbuminemia (HR = 1.745; 95% CI, 1.592–1.911; *p* < 0.001), and elevated CRP (HR = 1.389; 95% CI, 1.224–1.577; *p* < 0.001). BMI ≥30 kg/m^2^ and open-angle glaucoma were associated with smaller but statistically significant effects, whereas female sex remained protective (HR = 0.767; 95% CI, 0.699–0.841; *p* < 0.001).

For AKI, hypertension exerted the greatest effect (HR = 4.240; 95% CI, 3.226–5.573; *p* < 0.001), followed by reduced renal function at baseline (eGFR <60 mL/min/1.73 m^2^: HR = 3.539; 95% CI, 3.104–4.035; *p* < 0.001) and hypoalbuminemia (HR = 2.935; 95% CI, 2.595–3.320; *p* < 0.001). Diabetes (HR = 2.274; 95% CI, 1.995–2.593; *p* < 0.001), older age (HR = 1.656; 95% CI, 1.482–1.850; *p* < 0.001), and elevated CRP (HR = 1.944; 95% CI, 1.680–2.249; *p* < 0.001) were also significant predictors. Female sex again demonstrated a protective association (HR = 0.661; 95% CI, 0.589–0.742; *p* < 0.001), while BMI ≥ 30 kg/m^2^ showed no association, and open-angle glaucoma did not reach statistical significance (*p* = 0.058).

### 3.5. Sensitivity Analyses

Sensitivity analyses were performed to evaluate the robustness of the primary outcome findings under varying analytic conditions. In the first sensitivity analysis, outcome estimates were compared before and after propensity score matching. As shown in Supplementary Table S1, the direction and magnitude of associations between VDD and clinical outcomes remained consistent across analytic conditions. After matching, VDD continued to be associated with higher risks of all-cause mortality (HR = 1.104; 95% CI, 1.001–1.217; *p* = 0.048), MACE (HR = 1.151; 95% CI, 1.078–1.229; *p* < 0.001), and AKI (HR = 1.154; 95% CI, 1.056–1.261; *p* = 0.002), with no significant difference in renal function decline (*p* = 0.428).

In the second sensitivity analysis, alternative vitamin D thresholds were examined. As shown in Supplementary Table S2, defining VDD as 25(OH)D < 20 ng/mL yielded elevated risks for MACE (HR = 1.120; 95% CI, 1.014–1.235; *p* = 0.025) and AKI (HR = 1.237; 95% CI, 1.092–1.402; *p* = 0.001). The association with all-cause mortality demonstrated a similar trend (HR = 1.134; 95% CI, 0.990–1.299; *p* = 0.069), although not statistically significant. Across all analytic contrasts, no significant differences were observed in renal function decline (all *p* > 0.05).

Additional sensitivity analyses were conducted to assess residual confounding, exposure stability, outcome misclassification, and matching robustness. Negative control outcome analyses showed no significant associations with falsification endpoints (Supplementary Table S3), supporting the specificity of the primary findings. E-value analyses indicated moderate robustness of the associations for MACE and AKI to unmeasured confounding (Supplementary Table S4). Exclusion of patients with major acute illnesses around the time of vitamin D measurement yielded consistent or stronger associations for mortality, MACE, and AKI (Supplementary Table S5). Results were also consistent when propensity score matching was restricted to covariates with high data completeness (Supplementary Table S6). Finally, healthcare utilization analyses demonstrated a higher hospitalization burden in the VDD cohort despite similar ambulatory visit rates, providing contextual support for differential clinical risk rather than surveillance bias (Supplementary Table S7).

### 3.6. Competing Risk Analysis

Competing risk analyses using Aalen–Johansen estimators were conducted to evaluate the cumulative incidence of nonfatal clinical outcomes while accounting for death as a competing event (Supplementary Figure S1). At the 5- and 10-year follow-up horizons, the cumulative incidence of MACE was the highest among the evaluated outcomes in both the VDD and VDA cohorts, followed by AKI. The cumulative incidence of all-cause mortality was lower than that of nonfatal cardiorenal events but increased progressively over time. Renal function decline (eGFR < 60 mL/min/1.73 m^2^) demonstrated the lowest cumulative incidence throughout all follow-up periods. Overall, the Aalen–Johansen estimates showed similar between-group separation for MACE and AKI after accounting for death as a competing event, which is consistent with the interpretation that the primary associations were not explained solely by differential mortality censoring.

## 4. Discussion

In this large real-world cohort of patients with primary glaucoma, VDD was consistently associated with increased risks of all-cause mortality, MACE, and AKI, whereas long-term decline in renal function did not differ between exposure groups. These differences emerged within the first year of observation and persisted over a 5-year horizon. Subgroup findings further demonstrated that systemic risk factors—including impaired renal function, hypoalbuminemia, diabetes, hypertension, and elevated CRP—were strongly associated with long-term adverse outcomes, while sensitivity analyses confirmed the robustness of the results across analytic approaches and alternative definitions of VDD. Competing-risk modeling additionally showed that these associations were not attributable to differential mortality censoring. Taken together, the findings suggest that VDD is associated with a clinically vulnerable phenotype among patients with glaucoma.

The overall pattern of higher all-cause mortality, MACE, and AKI risk in VDD, alongside the absence of differences in incident eGFR < 60 mL/min/1.73 m^2^, may be contextualized by prior hypotheses regarding systemic and ocular roles of vitamin D, although these mechanisms were not directly examined in the present study. Vitamin D, through its active form 1,25-dihydroxyvitamin D, has been described as a pleiotropic steroid hormone capable of activating the VDR, a nuclear transcription factor expressed in immune cells, vascular endothelium, renal tubular epithelium, cardiomyocytes, and ocular tissues such as the retina, trabecular meshwork, and optic nerve head [30,31]. Several mechanistic pathways have been proposed in prior experimental and clinical studies and may help contextualize our observational findings, although none were directly examined in the present study.

Inflammation has been implicated in the pathogenesis and progression of ocular diseases, including glaucoma. Prior experimental and translational studies suggest that vitamin D signaling can modulate inflammatory pathways by downregulating NF-κB activation, suppressing Th1/Th17 polarization and proinflammatory cytokines such as IL-6 and TNF-α, restraining NLRP3 inflammasome activity, and promoting immune tolerance [32,33,34]. While our study did not directly assess inflammatory signaling, the strong associations observed between elevated CRP levels and adverse outcomes raise the possibility that VDD may reflect an underlying proinflammatory systemic milieu, rather than acting as a direct driver of inflammation. Accordingly, vitamin D deficiency in this context is more appropriately interpreted as a marker of inflammatory vulnerability rather than a direct mediator of inflammatory injury.

From a cardiovascular perspective, vitamin D has been proposed to influence vascular and myocardial function through effects on the renin–angiotensin–aldosterone system (RAAS) and endothelial homeostasis. Experimental and clinical data indicate that vitamin D signaling may suppress renin expression and attenuate downstream vasoconstriction, oxidative stress, and maladaptive myocardial remodeling, while VDR activation has been associated with enhanced endothelial nitric oxide bioavailability and antithrombotic, antifibrotic vascular effects [35,36,37]. Deficiency has therefore been hypothesized to coexist with endothelial dysfunction and RAAS activation—pathophysiological substrates relevant to myocardial infarction, heart failure, arrhythmia, and cerebrovascular events [38]. In our cohort, traditional cardiometabolic risk factors such as hypertension, diabetes, reduced eGFR, and hypoalbuminemia were dominant predictors of MACE, and VDD contributed a modest but consistent excess hazard. These findings are compatible with a vascular–inflammatory vulnerability framework, though they do not establish a causal cardiovascular mechanism attributable to vitamin D deficiency itself.

Renal susceptibility in the setting of VDD may be understood in a similar context. Prior studies have suggested that VDR signaling in renal tubular cells and podocytes may limit apoptosis, oxidative stress, and interstitial fibrosis, in part through modulation of TGF-β/SMAD pathways and intrarenal RAAS activity [39,40]. Vitamin D deficiency has therefore been proposed to be associated with reduced renal reserve and ischemic tolerance, potentially increasing susceptibility to AKI during acute systemic insults [41,42]. In our analysis, the highest AKI risks were observed among patients with established renal impairment, hypoalbuminemia, diabetes, hypertension, and elevated CRP, and VDD was associated with a modest excess hazard, particularly early after glaucoma diagnosis. In contrast, the absence of a difference in incident eGFR < 60 mL/min/1.73 m^2^ may reflect recovery following discrete AKI episodes, competing mortality risks, or the slowly progressive nature of CKD, for which a single baseline biomarker such as vitamin D is likely a weaker determinant than cumulative exposures including glycemic control, blood pressure, and nephrotoxin burden. The strong association between hypoalbuminemia and adverse outcomes further supports the interpretation of a malnutrition–inflammation–frailty phenotype overlapping with VDD.

The neurodegenerative characteristics of glaucoma offer additional context for the systemic vulnerability observed in this cohort. Experimental studies have demonstrated VDR expression in retinal ganglion cells and optic nerve head astrocytes, and vitamin D signaling has been shown to modulate oxidative stress, microglial activation, and extracellular matrix remodeling in ocular models [43,44,45]. However, it is important to emphasize that our study did not assess ophthalmic progression or local neurodegenerative pathways. Rather, glaucoma in this setting may represent an age-associated, comorbidity-rich condition in which systemic vascular, metabolic, and inflammatory susceptibilities converge. In this context, VDD is best interpreted as a biomarker of systemic vulnerability that co-segregates with adverse health trajectories, rather than as a mechanistic driver of disease.

It is essential to interpret these observational findings in the context of evidence from randomized controlled trials of vitamin D supplementation. Despite consistent associations between low vitamin D status and adverse cardiovascular, renal, and mortality outcomes in observational studies, large randomized trials and meta-analyses have frequently failed to demonstrate meaningful clinical benefit from vitamin D supplementation across diverse populations. Notably, major trials such as VITAL and ViDA did not show reductions in all-cause mortality or major cardiovascular events with routine vitamin D supplementation, even among individuals with low baseline vitamin D levels.

This apparent discrepancy between observational associations and interventional trial results reinforces the interpretation that vitamin D deficiency may function primarily as a marker of underlying systemic vulnerability, frailty, or chronic illness rather than a direct causal mediator of adverse outcomes. Accordingly, the present findings should not be interpreted as evidence supporting vitamin D supplementation as a therapeutic strategy to reduce mortality, cardiovascular events, or AKI in patients with glaucoma.

The clinical implications of these findings lie primarily in risk stratification rather than therapeutic intervention. Vitamin D status is a readily measurable laboratory parameter and may serve as an early indicator of systemic vulnerability in patients with glaucoma. The early divergence in AKI and MACE risks—detectable within the first year—suggests a potential window for proactive care focused on optimization of cardiometabolic risk factors, avoidance of nephrotoxins, and closer collaboration with primary care, nephrology, and cardiology services. Within this framework, vitamin D deficiency may help identify patients who would benefit from comprehensive evaluation and management of comorbid conditions rather than from vitamin D supplementation alone.

This study has several limitations inherent to observational research. Although extensive propensity score matching and sensitivity analyses were performed, residual and unmeasured confounding cannot be fully eliminated. In particular, important lifestyle and behavioral factors that are not routinely captured in electronic health records—such as nutritional status, sunlight exposure, physical activity, frailty or functional status, vitamin D supplementation, and health-seeking behaviors—may be unevenly distributed between exposure groups. These factors are closely related to both vitamin D status and systemic health outcomes and may partially or even largely explain the observed associations, thereby precluding causal inference. Vitamin D status was assessed using a single pre-diagnostic serum 25-hydroxyvitamin D measurement within 12 months prior to the index date, which may not fully reflect longitudinal variability or seasonal fluctuation. To mitigate misclassification, the measurement closest to the index date was used, and individuals with recent vitamin D status transitions were excluded; however, residual exposure misclassification remains possible. In addition, to reduce reverse causality related to acute illness-associated suppression of vitamin D levels, sensitivity analyses excluding patients with major acute events within 14 days of the vitamin D measurement were conducted, with associations for mortality, MACE, and AKI persisting and strengthening in the restricted cohort (Supplementary Table S5). Nevertheless, future studies incorporating repeated measurements or season-adjusted models would further strengthen exposure validity. Outcome ascertainment relied on diagnostic codes and available laboratory data, which may have resulted in under-recognition of mild or subclinical chronic kidney disease and misclassification of acute kidney injury severity. In addition, detailed indicators of glaucoma severity—such as intraocular pressure, visual field loss, or formal disease staging—were not available in the database, and glaucoma subtype based on diagnostic codes should therefore be interpreted as descriptive heterogeneity rather than a proxy for disease severity. Propensity score matching relied on available-case data, and several laboratory covariates had approximately 10–30% missingness (e.g., ferritin, C-reactive protein). If laboratory testing was performed differentially according to vitamin D status or underlying health conditions, selection bias cannot be fully excluded; however, the achievement of excellent post-matching covariate balance (SMD < 0.1 across variables) mitigates this concern. In addition, serum 25-hydroxyvitamin D testing was performed in only a subset of the overall glaucoma population and was not randomly ordered in routine clinical practice. Patients who underwent testing may therefore represent a higher-risk group with a greater comorbidity burden, limiting the generalizability of the findings to untested individuals. However, healthcare utilization analyses demonstrated that patients with vitamin D deficiency exhibited a greater inpatient burden despite similar outpatient visit rates compared with vitamin D-adequate patients (Supplementary Table S7), supporting the interpretation of vitamin D deficiency as a marker of increased clinical vulnerability rather than differential surveillance alone. Furthermore, although the use of a large federated health network enhances external validity, heterogeneity in population characteristics, healthcare practices, laboratory testing patterns (including vitamin D assessment), and access to care across regions may influence observed associations, warranting caution when extrapolating these findings to specific populations or healthcare settings. Finally, given the retrospective observational design, causality cannot be inferred, and whether modification of vitamin D status alters systemic risk profiles in patients with glaucoma remains to be determined. To further assess residual confounding and reverse causality, negative control outcome analyses and E-value sensitivity analyses were performed, supporting the interpretation of vitamin D deficiency as a marker of systemic vulnerability rather than a causal determinant, particularly for MACE and AKI outcomes.

## 5. Conclusions

Vitamin D deficiency was associated with higher risks of all-cause mortality, major adverse cardiovascular events, and acute kidney injury among patients with glaucoma. These associations emerged early during follow-up and remained consistent across landmark and sensitivity analyses, even after accounting for competing mortality. Given the observational nature of this study, causality cannot be inferred, and vitamin D deficiency should be interpreted as a marker of underlying systemic vulnerability rather than as a direct causal factor. Our findings support the consideration of vitamin D status as a marker for risk stratification in patients with glaucoma and underscore the need for further prospective studies to clarify its prognostic significance rather than therapeutic implications.

## Figures and Tables

**Table 1:** Baseline characteristics of glaucoma patients by vitamin D status, before and after propensity score matching.

Characteristics	Before Matching: 17,865 vs. 17,235				After Matching: 11,855 vs. 11,855			
	Mean ± SD	Patient Count	% of Cohort	Std. Diff.	Mean ± SD	Patient Count	% of Cohort	Std. Diff.
Demographics								
Age at Index	58.2 ± 12.8 vs. 64.5 ± 11.1	17,856 vs. 17,230	100% vs. 100%	0.522	61.9 ± 11.4 vs. 62.0 ± 11.5	11,855 vs. 11,855	100% vs. 100%	0.007
Female		11,812 vs. 12,273	66.2% vs. 71.2%	0.110		8163 vs. 8183	68.9% vs. 69.0%	0.004
Male		5966 vs. 4774	33.4% vs. 27.7%	0.124		3618 vs. 3593	30.5% vs. 30.3%	0.005
White		8933 vs. 11,470	50.0% vs. 66.6%	0.340		7048 vs. 7066	59.5% vs. 59.6%	0.003
Unknown Race		1642 vs. 1466	9.2% vs. 8.5%	0.024		1098 vs. 1124	9.3% vs. 9.5%	0.008
Not Hispanic or Latino		13,530 vs. 14,333	75.8% vs. 83.2%	0.184		9604 vs. 9600	81.0% vs. 81.0%	0.001
Hispanic or Latino		2786 vs. 1203	15.6% vs. 7.0%	0.275		1155 vs. 1132	9.7% vs. 9.5%	0.007
Black or African American		5322 vs. 2577	29.8% vs. 15.0%	0.362		2424 vs. 2429	20.4% vs. 20.5%	0.001
Asian		954 vs. 1091	5.3% vs. 6.3%	0.042		733 vs. 727	6.2% vs. 6.1%	0.002
Diagnosis								
Hypertensive diseases		9770 vs. 9063	54.7% vs. 52.6%	0.042		6199 vs. 6248	52.3% vs. 52.7%	0.008
Ischemic heart disease		2022 vs. 1774	11.3% vs. 10.3%	0.033		1270 vs. 1276	10.7% vs. 10.8%	0.002
Cerebrovascular diseases		1100 vs. 1062	6.2% vs. 6.2%	<0.001		703 vs. 716	5.9% vs. 6.0%	0.005
Diabetes mellitus		6408 vs. 4466	35.9% vs. 25.9%	0.217		3506 vs. 3516	29.6% vs. 29.7%	0.002
Medication								
Antilipidemic agents		6363 vs. 6230	35.6% vs. 36.2%	0.011		4148 vs. 4154	35.0% vs. 35.0%	0.001
Diuretics		5245 vs. 4219	29.4% vs. 24.5%	0.110		3116 vs. 3143	26.3% vs. 26.5%	0.005
Beta blockers		4645 vs. 4134	26.0% vs. 24.0%	0.047		2925 vs. 2913	24.7% vs. 24.6%	0.002
ACE inhibitors		4038 vs. 2980	22.6% vs. 17.3%	0.133		2260 vs. 2287	19.1% vs. 19.3%	0.006
Calcium channel blockers		3666 vs. 3038	20.5% vs. 17.6%	0.074		2194 vs. 2190	18.5% vs. 18.5%	0.001
Angiotensin II inhibitors		2253 vs. 2321	12.6% vs. 13.5%	0.025		1522 vs. 1549	12.8% vs. 13.1%	0.007
Blood glucose regulation agents		5827 vs. 3906	32.6% vs. 22.7%	0.224		3122 vs. 3094	26.3% vs. 26.1%	0.005
Laboratory (Blood test)								
Calcidiol	19.6 ± 6.6 vs. 43.0 ± 12.6	16,545 vs. 16,156	92.7% vs. 93.8%	2.331	20.4 ± 6.4 vs. 42.6 ± 12.7	10,877 vs. 11,081	91.8% vs. 93.5%	2.206
Creatinine	1.1 ± 1.9 vs. 1.1 ± 2.6	15,726 vs. 15,290	88.1% vs. 88.7%	0.022	1.0 ± 2.0 vs. 1.1 ± 2.9	10,364 vs. 10,389	87.4% vs. 87.6%	0.024
Urea nitrogen	17.5 ± 11.5 vs. 17.9 ± 9.4	15,905 vs. 15,296	89.1% vs. 88.8%	0.040	17.3 ± 10.1 vs. 17.8 ± 9.9	10,416 vs. 10,437	87.9% vs. 88.0%	0.048
Bicarbonate	26.5 ± 3.1 vs. 26.9 ± 2.9	15,832 vs. 15,232	88.7% vs. 88.4%	0.142	26.6 ± 3.0 vs. 26.8 ± 3.0	10,373 vs. 10,391	87.5% vs. 87.7%	0.056
Sodium	139.2 ± 2.8 vs. 139.6 ± 2.9	15,891 vs. 15,287	89.0% vs. 88.7%	0.117	139.4 ± 2.8 vs. 139.5 ± 2.8	10,413 vs. 10,433	87.8% vs. 88.0%	0.042
Potassium	4.2 ± 0.4 vs. 4.2 ± 0.4	15,920 vs. 15,319	89.2% vs. 88.9%	0.092	4.2 ± 0.4 vs. 4.2 ± 0.4	10,432 vs. 10,450	88.0% vs. 88.1%	0.017
Glucose	121.1 ± 58.8 vs. 109.0 ± 39.4	15,802 vs. 15,191	88.5% vs. 88.2%	0.243	114.1 ± 49.5 vs. 111.9 ± 43.4	10,346 vs. 10,381	87.3% vs. 87.6%	0.046
0–60 mg/dL		662 vs. 378	3.7% vs. 2.2%	0.090		312 vs. 312	2.6% vs. 2.6%	<0.001
60–90 mg/dL		6714 vs. 6348	37.6% vs. 36.8%	0.016		4331 vs. 4309	36.5% vs. 36.3%	0.004
90–120 mg/dL		10,395 vs. 10,728	58.2% vs. 62.3%	0.083		7121 vs. 7077	60.1% vs. 59.7%	0.008
120–150 mg/dL		5324 vs. 4065	29.8% vs. 23.6%	0.141		3052 vs. 3116	25.7% vs. 26.3%	0.012
150–180 mg/dL		3673 vs. 2436	20.6% vs. 14.1%	0.170		1984 vs. 1975	16.7% vs. 16.7%	0.002
Calcium	9.3 ± 0.6 vs. 9.5 ± 0.5	15,883 vs. 15,374	89.0% vs. 89.2%	0.237	9.4 ± 0.5 vs. 9.4 ± 0.5	10,426 vs. 10,470	87.9% vs. 88.3%	0.076
0–8.50 mg/dL		2417 vs. 1353	13.5% vs. 7.9%	0.185		1126 vs. 1126	9.5% vs. 9.5%	<0.001
8.50–10 mg/dL		14,686 vs. 13,981	82.2% vs. 81.1%	0.029		9592 vs. 9609	80.9% vs. 81.1%	0.004
10–11 mg/dL		2943 vs. 3670	16.5% vs. 21.3%	0.123		2201 vs. 2167	18.6% vs. 18.3%	0.007
11–13 mg/dL		258 vs. 228	1.4% vs. 1.3%	0.010		163 vs. 158	1.4% vs. 1.3%	0.004
Magnesium	2.0 ± 0.3 vs. 2.0 ± 0.3	3382 vs. 2834	18.9% vs. 16.4%	0.063	2.0 ± 0.3 vs. 2.0 ± 0.3	1986 vs. 2005	16.8% vs. 16.9%	0.030
Phosphate	3.7 ± 0.9 vs. 3.5 ± 0.8	3984 vs. 3231	22.3% vs. 18.8%	0.137	3.6 ± 0.8 vs. 3.5 ± 0.8	2312 vs. 2332	19.5% vs. 19.7%	0.032
Leukocytes	8.9 ± 90.1 vs. 11.1 ± 139.5	12,301 vs. 11,251	68.9% vs. 65.3%	0.018	9.2 ± 98.6 vs. 11.9 ± 150.5	7815 vs. 7842	65.9% vs. 66.1%	0.021
Hemoglobin	13.1 ± 1.9 vs. 13.3 ± 1.6	14,411 vs. 13,486	80.7% vs. 78.3%	0.143	13.2 ± 1.7 vs. 13.2 ± 1.7	9363 vs. 9353	79.0% vs. 78.9%	0.038
Hematocrit	39.5 ± 5.2 vs. 40.2 ± 4.6	14,597 vs. 13,849	81.7% vs. 80.4%	0.133	39.9 ± 4.9 vs. 40.1 ± 4.7	9532 vs. 9505	80.4% vs. 80.2%	0.032
Platelets	245.2 ± 77.2 vs. 239.3 ± 69.5	14,326 vs. 13,551	80.2% vs. 78.6%	0.080	242.2 ± 73.6 vs. 241.1 ± 70.9	9326 vs. 9291	78.7% vs. 78.4%	0.015
Alanine aminotransferase	26.4 ± 37.1 vs. 24.6 ± 27.8	14,560 vs. 14,129	81.5% vs. 82.0%	0.054	25.2 ± 42.0 vs. 25.4 ± 32.4	9578 vs. 9584	80.8% vs. 80.8%	0.006
Aspartate aminotransferase	26.0 ± 115.4 vs. 25.0 ± 28.7	14,460 vs. 14,027	81.0% vs. 81.4%	0.012	25.9 ± 141.5 vs. 25.3 ± 33.9	9506 vs. 9500	80.2% vs. 80.1%	0.006
Alkaline phosphatase	84.4 ± 48.0 vs. 75.6 ± 33.5	14,176 vs. 13,677	79.4% vs. 79.4%	0.215	80.0 ± 41.5 vs. 78.4 ± 35.4	9300 vs. 9283	78.4% vs. 78.3%	0.042
0–50 U/L		1674 vs. 2244	9.4% vs. 13.0%	0.116		1293 vs. 1319	10.9% vs. 11.1%	0.007
50–70 U/L		5329 vs. 6081	29.8% vs. 35.3%	0.116		3852 vs. 3810	32.5% vs. 32.1%	0.008
70–90 U/L		5765 vs. 5167	32.3% vs. 30.0%	0.050		3673 vs. 3675	31.0% vs. 31.0%	<0.001
90–120 U/L		4298 vs. 3069	24.1% vs. 17.8%	0.154		2414 vs. 2422	20.4% vs. 20.4%	0.002
Bilirubin, total	0.6 ± 0.5 vs. 0.6 ± 0.4	14,102 vs. 13,597	79.0% vs. 78.9%	0.022	0.6 ± 0.4 vs. 0.6 ± 0.4	9248 vs. 9227	78.0% vs. 77.8%	0.020
Albumin	4.0 ± 0.5 vs. 4.1 ± 0.4	14,137 vs. 13,649	79.2% vs. 79.2%	0.221	4.0 ± 0.5 vs. 4.1 ± 0.4	9265 vs. 9280	78.2% vs. 78.3%	0.031
0–3 g/dL		1384 vs. 699	7.8% vs. 4.1%	0.157		609 vs. 603	5.1% vs. 5.1%	0.002
3–4 g/dL		7964 vs. 6782	44.6% vs. 39.4%	0.106		4822 vs. 4862	40.7% vs. 41.0%	0.007
4–5 g/dL		9636 vs. 10,386	54.0% vs. 60.3%	0.128		6763 vs. 6810	57.0% vs. 57.4%	0.008
Total protein	7.1 ± 0.7 vs. 7.0 ± 0.6	13,930 vs. 13,377	78.0% vs. 77.6%	0.140	7.1 ± 0.7 vs. 7.1 ± 0.6	9111 vs. 9084	76.9% vs. 76.6%	0.029
Total Cholesterol	183.3 ± 50.0 vs. 180.1 ± 46.5	12,806 vs. 12,588	71.7% vs. 73.1%	0.066	182.2 ± 48.7 vs. 181.1 ± 47.7	8503 vs. 8416	71.7% vs. 71.0%	0.023
0–150 mg/dL		3175 vs. 3200	17.8% vs. 18.6%	0.021		2132 vs. 2138	18.0% vs. 18.0%	0.001
150–200 mg/dL		6276 vs. 6415	35.1% vs. 37.2%	0.043		4220 vs. 4148	35.6% vs. 35.0%	0.013
200–300 mg/dL		4969 vs. 4554	27.8% vs. 26.4%	0.031		3166 vs. 3210	26.7% vs. 27.1%	0.008
Cholesterol in LDL	105.3 ± 39.8 vs. 100.3 ± 36.4	12,698 vs. 12,533	71.1% vs. 72.7%	0.132	103.6 ± 38.5 vs. 102.2 ± 37.8	8461 vs. 8367	71.4% vs. 70.6%	0.037
0–50 mg/dL		937 vs. 864	5.2% vs. 5.0%	0.011		600 vs. 610	5.1% vs. 5.1%	0.004
50–100 mg/dL		5595 vs. 6306	31.3% vs. 36.6%	0.111		3946 vs. 3942	33.3% vs. 33.3%	0.001
100–150 mg/dL		6043 vs. 5746	33.8% vs. 33.3%	0.010		3959 vs. 3908	33.4% vs. 33.0%	0.009
Cholesterol in HDL	50.6 ± 18.4 vs. 55.2 ± 21.2	12,794 vs. 12,592	71.7% vs. 73.1%	0.235	52.7 ± 19.2 vs. 53.2 ± 20.3	8500 vs. 8423	71.7% vs. 71.1%	0.027
0–40 mg/dL		3634 vs. 2615	20.4% vs. 15.2%	0.136		2003 vs. 2027	16.9% vs. 17.1%	0.005
40–60 mg/dL		6827 vs. 6203	38.2% vs. 36.0%	0.046		4401 vs. 4383	37.1% vs. 37.0%	0.003
60–80 mg/dL		2940 vs. 3841	16.5% vs. 22.3%	0.148		2295 vs. 2288	19.4% vs. 19.3%	0.001
Triglyceride	141.6 ± 116.1 vs. 119.3 ± 83.6	12,575 vs. 12,348	70.4% vs. 71.7%	0.221	131.2 ± 98.7 vs. 126.1 ± 91.7	8386 vs. 8254	70.7% vs. 69.6%	0.053
0–100 mg/dL		5349 vs. 6408	30.0% vs. 37.2%	0.154		3940 vs. 3900	33.2% vs. 32.9%	0.007
100–150 mg/dL		4347 vs. 4258	24.3% vs. 24.7%	0.009		2867 vs. 2874	24.2% vs. 24.2%	0.001
150–200 mg/dL		2474 vs. 2130	13.9% vs. 12.4%	0.044		1537 vs. 1557	13.0% vs. 13.1%	0.005
200–300 mg/dL		1912 vs. 1390	10.7% vs. 8.1%	0.091		1075 vs. 1086	9.1% vs. 9.2%	0.003
Hemoglobin A1c	6.9 ± 2.0 vs. 6.3 ± 1.5	9969 vs. 8215	55.8% vs. 47.7%	0.314	6.5 ± 1.7 vs. 6.4 ± 1.6	6032 vs. 5914	50.9% vs. 49.9%	0.050
0–5%		555 vs. 487	3.1% vs. 2.8%	0.017		352 vs. 347	3.0% vs. 2.9%	0.002
5–6%		4214 vs. 4342	23.6% vs. 25.2%	0.037		2885 vs. 2874	24.3% vs. 24.2%	0.002
6–7%		3622 vs. 3194	20.3% vs. 18.5%	0.044		2324 vs. 2366	19.6% vs. 20.0%	0.009
7–8%		2091 vs. 1545	11.7% vs. 9.0%	0.090		1231 vs. 1224	10.4% vs. 10.3%	0.002
8–9%		1328 vs. 805	7.4% vs. 4.7%	0.116		701 vs. 684	5.9% vs. 5.8%	0.006
≥9%		1842 vs. 656	10.3% vs. 3.8%	0.256		609 vs. 604	5.1% vs. 5.1%	0.002
Iron	70.6 ± 41.5 vs. 77.7 ± 36.9	3116 vs. 2431	17.5% vs. 14.1%	0.180	75.3 ± 41.5 vs. 75.1 ± 37.1	1796 vs. 1802	15.1% vs. 15.2%	0.005
0–50 µg/dL		1219 vs. 673	6.8% vs. 3.9%	0.130		565 vs. 566	4.8% vs. 4.8%	<0.001
50–100 µg/dL		1770 vs. 1475	9.9% vs. 8.6%	0.047		1076 vs. 1071	9.1% vs. 9.0%	0.001
100–200 µg/dL		606 vs. 634	3.4% vs. 3.7%	0.015		419 vs. 441	3.5% vs. 3.7%	0.010
Ferritin	245.7 ± 547.5 vs. 201.2 ± 551.2	3254 vs. 2474	18.2% vs. 14.4%	0.081	213.2 ± 427.3 vs. 202.3 ± 485.9	1871 vs. 1882	15.8% vs. 15.9%	0.024
CRP	18.4 ± 37.8 vs. 11.6 ± 28.5	2156 vs. 2050	12.1% vs. 11.9%	0.203	13.9 ± 31.2 vs. 13.6 ± 31.4	1354 vs. 1363	11.4% vs. 11.5%	0.009
0–10 mg/L		1486 vs. 1657	8.3% vs. 9.6%	0.045		1040 vs. 1049	8.8% vs. 8.8%	0.003
10–20 mg/L		488 vs. 327	2.7% vs. 1.9%	0.056		268 vs. 257	2.3% vs. 2.2%	0.006
20–40 mg/L		315 vs. 201	1.8% vs. 1.2%	0.050		151 vs. 165	1.3% vs. 1.4%	0.010

Abbreviations: CRP, C-reactive protein; HDL, high-density lipoprotein; LDL, low-density lipoprotein; SD, standard deviation; Std. Diff., standardized difference.

**Table 2:** Landmark analyses of clinical outcomes at 1, 3, and 5 years.

Follow-Up	Outcomes	Cohorts	Patients in Cohort	Patients with Outcome	Survival Probability	Hazard Ratio	95% CI	Log-Rank Test, *p*-Value
1 year	All-cause mortality	VDD	10,033	214	97.82%	1.306	(1.066–1.600)	0.010
		VDA	10,012	165	98.33%			
3 years		VDD	10,029	512	94.61%	1.132	(0.998–1.284)	0.053
		VDA	10,014	460	95.19%			
5 years		VDD	11,040	833	91.81%	1.104	(1.001–1.217)	0.048
		VDA	11,023	770	92.52%			
1 year	MACE	VDD	7286	550	92.29%	1.278	(1.127–1.449)	<0.001
		VDA	7320	439	93.90%			
3 years		VDD	7258	1221	82.27%	1.172	(1.080–1.272)	<0.001
		VDA	7318	1080	84.54%			
5 years		VDD	7984	1890	74.42%	1.151	(1.078–1.229)	<0.001
		VDA	8002	1703	77.20%			
1 year	AKI	VDD	9762	287	97.00%	1.288	(1.081–1.534)	0.004
		VDA	9721	224	97.66%			
3 years		VDD	9768	638	93.10%	1.113	(0.995–1.246)	0.061
		VDA	9721	581	93.74%			
5 years		VDD	10,683	1036	89.42%	1.154	(1.056–1.261)	0.002
		VDA	10,622	915	90.74%			
1 year	eGFR < 60 mL/min/1.73 m^2^	VDD	6745	473	92.85%	0.999	(0.879–1.137)	0.993
		VDA	6458	455	92.85%			
3 years		VDD	6759	1083	83.09%	1.050	(0.963–1.144)	0.271
		VDA	6456	995	83.93%			
5 years		VDD	7284	1581	76.59%	0.972	(0.907–1.042)	0.428
		VDA	6997	1576	75.97%			

Abbreviations: AKI, acute kidney injury; CI, confidence interval; eGFR, estimated glomerular filtration rate; MACE, major adverse cardiovascular events; VDA, vitamin D adequacy; VDD, vitamin D deficiency.

## Data Availability

The data presented in this study are available upon request from the corresponding author due to privacy and ethical restrictions. The dataset was obtained from the TriNetX global federated health research network, which aggregates deidentified electronic medical records from multiple healthcare institutions. Access to the dataset is restricted by institutional policies and data-sharing agreements. Researchers interested in accessing the data may request it from TriNetX, subject to institutional approval and compliance with data privacy regulations.

## Supplementary Materials

The following supporting information can be downloaded at: https://www.mdpi.com/article/10.3390/nu18020261/s1, Table S1. Five-year outcomes before and after propensity score matching. Table S2. Five-year adverse outcomes using alternative vitamin D deficiency thresholds. Table S3. Negative Control Outcome Analyses for Acute Appendicitis and Inguinal Hernia According to Vitamin D Status. Table S4. E-Value Sensitivity Analysis for Primary Cardiorenal Outcomes by Vitamin D Status. Table S5. Sensitivity Analysis of Kaplan–Meier Survival Estimates Before and After Exclusion of Acute Illness Around Vitamin D Measurement. Table S6. Sensitivity Analysis Using Full versus Restricted Propensity Score Matching. Table S7. Healthcare Utilization in Matched Glaucoma Cohorts by Vitamin D Status. Figure S1. Aalen–Johansen Cumulative Incidence Curves for Competing Risk Analyses.

## Abbreviations

The following abbreviations are used in this manuscript:

25(OH)D	25-Hydroxyvitamin D
ACEI	Angiotensin-Converting Enzyme Inhibitor
AKI	Acute Kidney Injury
ALP	Alkaline Phosphatase
ALT	Alanine Aminotransferase
ARB	Angiotensin II Receptor Blocker
AST	Aspartate Aminotransferase
CKD	Chronic Kidney Disease
CRP	C-Reactive Protein
DM	Diabetes Mellitus
eGFR	Estimated Glomerular Filtration Rate
HbA1c	Hemoglobin A1c
HDL	High-Density Lipoprotein
LDL	Low-Density Lipoprotein
MACE	Major Adverse Cardiovascular Events
MDRD	Modification of Diet in Renal Disease
NLRP3	NOD-, LRR- and Pyrin Domain-Containing Protein 3 (Inflammasome)
POAG	Primary Open-Angle Glaucoma
PACG	Primary Angle-Closure Glaucoma
RAAS	Renin–Angiotensin–Aldosterone System
VDA	Vitamin D Adequacy
VDD	Vitamin D Deficiency
VDR	Vitamin D Receptor
