# Supplementary material for: Association Between Vitamin D Deficiency and Systemic Outcomes in Patients with Glaucoma: A Real-World Cohort Study
Source: Nutrients. 2026 Jan 14;18(2):261. doi: 10.3390/nu18020261 (PMC12844972; doi:10.3390/nu18020261)
Supplement: Supplementary file 1 [file nutrients-18-00261-s001.zip › nutrients-4018054-supplementary.pdf]

Supplementary materials

Table S1. Five-year outcomes before and after propensity score matching.

Table S2. Five-year adverse outcomes using alternative vitamin D deficiency thresholds.

Table S3. Negative Control Outcome Analyses for Acute Appendicitis and Inguinal Hernia According to Vitamin D Status.

Table S4. E-Value Sensitivity Analysis for Primary Cardiorenal Outcomes by Vitamin D Status.

Table S5. Sensitivity Analysis of Kaplan–Meier Survival Estimates Before and After Exclusion of Acute Illness Around Vitamin D Measurement.

Table S6. Sensitivity Analysis Using Full versus Restricted Propensity Score Matching.

Table S7. Healthcare Utilization in Matched Glaucoma Cohorts by Vitamin D Status.

Figure S1. Aalen–Johansen Cumulative Incidence Curves for Competing Risk Analyses.

Supplementary Table S1. Five-year outcomes before and after propensity score matching. Sensitivity analysis comparing 5-year clinical outcomes before and after propensity score matching among glaucoma patients with vitamin D deficiency (VDD) versus adequacy (VDA). Outcomes include all-cause mortality, major adverse cardiovascular events (MACE), acute kidney injury (AKI), and renal function decline (eGFR <60 mL/min/1.73 m^2). Values shown include cohort size, event counts, Kaplan–Meier survival probability at 5 years, hazard ratios with 95% confidence intervals, and log-rank test p-values.

PSM Cohort	Outcomes	Cohorts	Patients in cohort	Patients with outcome	Survival probability	Hazard Ratio	95% CI	Log-Rank test p value
Before	All-cause mortality	VDD	15,328	1,155	91.61%	1.066	(0.981–1.159)	0.131
		VDA	14,438	1,070	92.05%			
After		VDD	11,040	833	91.81%	1.104	(1.001–1.217)	0.048
		VDA	11,023	770	92.52%			
Before	MACE	VDD	11,105	2,484	75.27%	1.017	(0.962–1.076)	0.548
		VDA	10,477	2,406	75.37%			
After		VDD	7,984	1,890	74.42%	1.151		< 0.001

		VDA	8,002	1,703	77.20%		(1.078–1.229)	
Before	AKI	VDD	14,575	1,469	88.79%	1.242	(1.151–1.340)	< 0.001
		VDA	14,226	1,213	90.82%			
After		VDD	10,683	1,036	89.42%	1.154	(1.056–1.261)	0.002
		VDA	10,622	915	90.74%			
Before	eGFR <60 mL/min/1.73 m^2	VDD	10,374	2,113	77.52%	0.860	(0.810–0.913)	< 0.001
		VDA	9,150	2,199	74.30%			
After		VDD	7,284	1,581	76.59%	0.972	(0.907–1.042)	0.428
		VDA	6,997	1,576	75.97%			

AKI, acute kidney injury; CI, confidence interval; eGFR, estimated glomerular filtration rate; MACE, major adverse cardiovascular events; PSM, propensity score matching; VDA, vitamin D adequacy; VDD, vitamin D deficiency.

Supplementary Table S2. Five-year adverse outcomes using alternative vitamin D deficiency thresholds. Comparisons include 25(OH)D <20 ng/mL vs ≥30 ng/mL and 25(OH)D <30 ng/mL vs ≥30 ng/mL. Outcomes assessed were all-cause mortality, major adverse cardiovascular events (MACE), acute kidney injury (AKI), and renal function decline (eGFR <60 mL/min/1.73 m^2). Values include cohort size, number of events, Kaplan–Meier survival probability, hazard ratios with 95% confidence intervals, and log-rank p-values.

Comparison	Outcomes	Cohorts	Patients in cohort	Patients with outcome	Survival probability	Hazard Ratio	95% CI	Log-Rank test p value
25(OH)D < 20 vs ≥ 30 ng/mL	All-cause mortality	VDD	5,097	435	90.46%	1.134	(0.990–1.299)	0.069
		VDA	5,133	399	91.53%			
25(OH)D < 20 vs ≥ 30 ng/mL		VDD	11,040	833	91.81%	1.104	(1.001–1.217)	0.048
		VDA	11,023	770	92.52%			
25(OH)D < 20 vs ≥ 30 ng/mL	MACE	VDD	3,650	817	75.17%	1.120	1.014–1.235	0.025
		VDA	3,690	767	77.32%			
		VDD	7,984	1,890	74.42%	1.151		< 0.001

25(OH)D < 20 vs ≥ 30 ng/mL		VDA	8,002	1,703	77.20%		(1.078–1.229)	
25(OH)D < 20 vs ≥ 30 ng/mL	AKI	VDD	4,877	542	87.59%	1.237	(1.092–1.402)	0.001
		VDA	4,837	451	89.81%			
25(OH)D < 20 vs ≥ 30 ng/mL		VDD	10,683	1,036	89.42%	1.154	(1.056–1.261)	0.002
		VDA	10,622	915	90.74%			
25(OH)D < 20 vs ≥ 30 ng/mL	eGFR <60 mL/min/1.73 m^2	VDD	3,466	757	75.84%	1.023	(0.923–1.133)	0.664
		VDA	3,255	714	76.17%			
25(OH)D < 20 vs ≥ 30 ng/mL		VDD	7,284	1,581	76.59%	0.972	(0.907–1.042)	0.428
		VDA	6,997	1,576	75.97%			

Abbreviations: AKI, acute kidney injury; CI, confidence interval; eGFR, estimated glomerular filtration rate; MACE, major adverse cardiovascular events; VDA, vitamin D adequacy; VDD, vitamin D deficiency.

Supplementary Table S3. Negative Control Outcome Analyses for Acute Appendicitis and Inguinal Hernia According to Vitamin D Status. Negative control outcome analyses comparing patients with vitamin D deficiency (VDD) and vitamin D adequacy (VDA). Acute appendicitis (ICD-10-CM K35.x) and inguinal hernia (ICD-10-CM K40.x) were used as falsification endpoints because they lack plausible biological links to vitamin D status or the cardiorenal pathways studied. Acute appendicitis served as an acute-event control, while inguinal hernia functioned as a proxy for healthcare utilization and detection bias. Time-to-event analyses were performed using Kaplan–Meier methods and Cox proportional hazards models. The absence of significant associations supports the specificity of the primary findings and does not suggest substantial residual confounding.

Outcomes	Cohorts	Patients in Cohort	Patients with Outcome	Survival Probability at End of Time Window	Hazard Ratio	95% CI	Log-Rank Test p value
Acute appendicitis	VDD	10,271	26	99.72%	0.759	0.457, 1.261	0.2853
	VDA	10,272	35	99.62%			
Inguinal hernia	VDD	10,127	151	98.32%	0.915	0.734, 1.14	0.4263
	VDA	10,113	168	98.17%			

Abbreviations: CI, confidence interval; VDA, vitamin D adequacy; VDD, vitamin D deficiency.

Supplementary Table S4. E-Value Sensitivity Analysis for Primary Cardiorenal Outcomes by Vitamin D Status. Larger E-values indicate greater robustness to unmeasured confounding; however, causal inference cannot be established. Associations for MACE and AKI demonstrated moderate robustness, whereas the mortality association was more susceptible to residual confounding.

Outcomes	Cohorts	Hazard Ratio	95% CI	Hazard Ratio E-value	CI E-value
All-cause mortality	VDD<br>VDA	1.104	(1.001–1.217)	1.44	1.03
MACE	VDD<br>VDA	1.151	(1.078–1.229)	1.57	1.37
AKI	VDD<br>VDA	1.154	(1.056–1.261)	1.58	1.30
eGFR <60 mL/min/1.73 m^2	VDD<br>VDA	0.972	(0.907–1.042)	1.20	1.00

Abbreviations: AKI, acute kidney injury; CI, confidence interval; eGFR, estimated glomerular filtration rate; MACE, major adverse cardiovascular events; VDA, vitamin D adequacy; VDD, vitamin D deficiency.

Supplementary Table S5. Sensitivity Analysis of Kaplan–Meier Survival Estimates Before and After Exclusion of Acute Illness Around Vitamin D Measurement. Kaplan–Meier survival analyses comparing patients with vitamin D deficiency (VDD) and vitamin D adequacy (VDA) in the full cohort and in a restricted cohort excluding individuals with major acute illnesses within 14 days of the 25-hydroxyvitamin D measurement. Acute illnesses included sepsis, pneumonia, heart failure, and acute myocardial infarction. Hazard ratios, 95% confidence intervals, and log-rank p-values are shown for all-cause mortality, major adverse cardiovascular events (MACE), acute kidney injury (AKI), and incident eGFR <60 mL/min/1.73 m^2.

**KM-survival analysis**		**Full cohort comparison**			**Exclude acute illness comparison**		
**Outcomes**	**Cohorts**	**Hazard Ratio**	**95% CI**	**Log-Rank Test p-value**	**Hazard Ratio**	**95% CI**	**Log-Rank Test p-value**
All-cause mortality	VDD	1.104	(1.001–1.217)	0.048	1.465	(1.254–1.712)	<0.001
	VDA						
MACE	VDD	1.151	(1.078–1.229)	<0.001	1.268	(1.137–1.415)	<0.001
	VDA						
AKI	VDD	1.154	(1.056–1.261)	0.002	1.219	(1.050–1.415)	0.009
	VDA						

eGFR <60 mL/min/1.73 m^2	VDD	0.972	(0.907–1.042)	0.428	0.894	(0.796–1.004)	0.058
	VDA						

Abbreviations: AKI, acute kidney injury; CI, confidence interval; eGFR, estimated glomerular filtration rate; MACE, major adverse cardiovascular events; VDA, vitamin D adequacy; VDD, vitamin D deficiency.

Supplementary Table S6. Sensitivity Analysis Using Full versus Restricted Propensity Score Matching. Primary outcomes comparing vitamin D deficiency (VDD) and vitamin D adequacy (VDA) using two propensity score matching approaches: a full model with 47 covariates and a restricted model with 27 covariates including only laboratory variables with high completeness. Hazard ratios, 95% confidence intervals, and log-rank p-values are shown. Consistent results across models indicate stability of the observed associations across different matching specifications.

Outcomes	Cohorts	PSM with 47 covariates (27 Labs)			PSM with 27 covariates (7 Labs)		
		**Hazard Ratio**	**95% CI**	**Log-Rank Test p-value**	**Hazard Ratio**	**95% CI**	**Log-Rank Test p-value**
All-cause mortality	VDD	1.104	(1.001–1.217)	0.048	1.141	(1.030–1.264)	0.012
	VDA						
MACE	VDD	1.151	(1.078–1.229)	<0.001	1.208	(1.128–1.294)	<0.001
	VDA						
AKI	VDD	1.154	(1.056–1.261)	0.002	1.177	(1.074–1.289)	<0.001
	VDA						
eGFR <60 mL/min/1.73 m^2	VDD	0.972	(0.907–1.042)	0.428	1.027	(0.955–1.105)	0.473
	VDA						

Abbreviations: AKI, acute kidney injury; CI, confidence interval; eGFR, estimated glomerular filtration rate; MACE, major adverse cardiovascular events; VDA, vitamin D adequacy; VDD, vitamin D deficiency.

Supplementary Table S7. Healthcare Utilization in Matched Glaucoma Cohorts by Vitamin D Status. Healthcare utilization over 5-year follow-up comparing matched patients with vitamin D deficiency (VDD) and vitamin D adequacy (VDA). Outcomes include ambulatory visits and hospitalizations. Values are presented as the proportion of patients with ≥1 event and the mean number of events per patient. These analyses were conducted to help contextualize potential outcome misclassification and to assess whether differences in clinical events may be reflected in overall healthcare utilization patterns.

Outcomes	Cohorts	Patients in cohort	Patients with outcome	Mean	Standard Deviation	p-value
Ambulatory visit	VDD	4,494	4,186	47.527	55.385	<0.001
	VDA	4,494	4,193	57.877	62.835	
Hospitalization	VDD	4,494	1,668	1.821	4.994	0.003
	VDA	4,494	1,464	1.520	4.644	
Ophthalmology service and procedure	VDD	4,494	2,219	2.366	4.439	<0.001
	VDA	4,494	2,173	2.186	4,841	

Abbreviations: CI, confidence interval; VDA, vitamin D adequacy; VDD, vitamin D deficiency.

Supplementary Figure S1. Aalen–Johansen Cumulative Incidence Curves for Competing Risk Analyses. Cumulative incidence curves depict the 5- and 10-year cumulative incidence of all-cause mortality, major adverse cardiovascular events (MACE), acute kidney injury (AKI), and renal function decline (eGFR <60 mL/min/1.73 m^2) among glaucoma patients with vitamin D deficiency (VDD) and vitamin D adequacy (VDA). The Aalen–Johansen estimator was applied to account for death as a competing event for nonfatal outcomes. Panels A–B display 5-year cumulative incidence for VDD and VDA, and Panels C–D present the corresponding 10-year estimates.

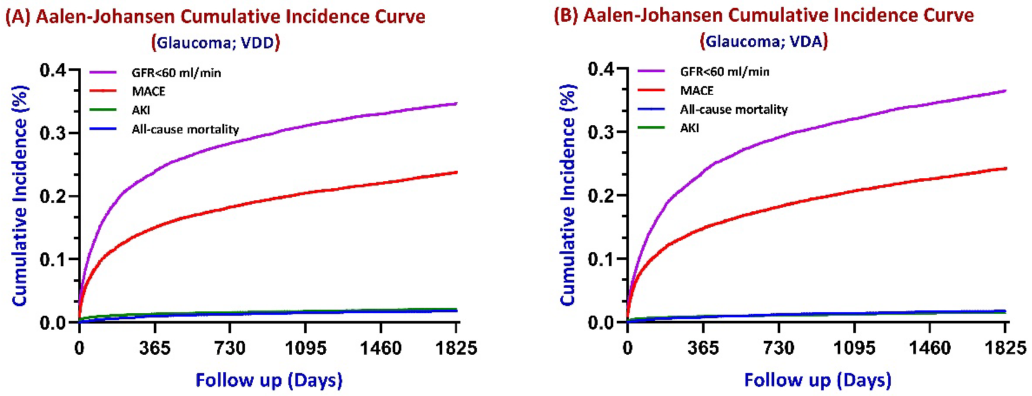

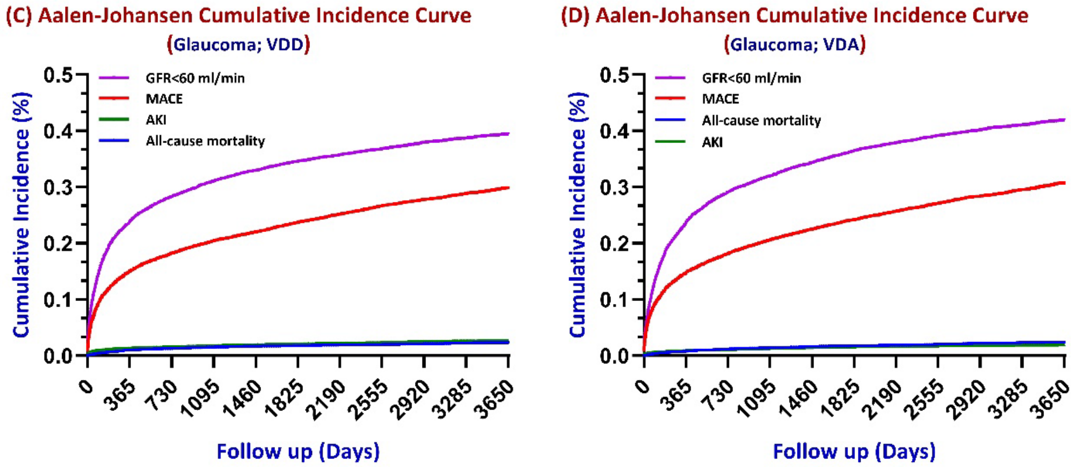

(C) Aalen-Johansen Cumulative Incidence Curve
(Glaucoma; VDD)
Cumulative Incidence (%)
0.5
0.4
0.3
0.2
0.1
0.0
GFR<60 ml/min
MACE
AKI
All-cause mortality
0
365
730
1095
1460
1825
2190
2555
2920
3285
3650
Follow up (Days)
(D) Aalen-Johansen Cumulative Incidence Curve
(Glaucoma; VDA)
Cumulative Incidence (%)
0.5
0.4
0.3
0.2
0.1
0.0
GFR<60 ml/min
MACE
All-cause mortality
AKI
0
365
730
1095
1460
1825
2190
2555
2920
3285
3650
Follow up (Days)
